# Community-Acquired Acinetobacter radioresistens Bacteremia in an Immunocompetent Host

**DOI:** 10.7759/cureus.29650

**Published:** 2022-09-27

**Authors:** Artemii Lazarev, Jane Hyun, Jacob L Sanchez, Larissa Verda

**Affiliations:** 1 Internal Medicine, Mount Sinai Hospital, Chicago, USA; 2 Internal Medicine, Ross University School of Medicine, Miramar, USA

**Keywords:** acinetobacter baumannii, carbapenem resistance, maldi-tof, bacteremia in immune-competent individuals, acinetobacter, carbapenemase, pneumonia, acinetobacter radioresistens, multidrug-resistant acinetobacter, bacteremia

## Abstract

*Acinetobacter* species are gram-negative coccobacilli ubiquitous in nature and widely distributed in the environment. *Acinetobacter baumannii* is a bacteria commonly seen in the hospital setting, responsible for causing a wide range of bloodstream infections, urinary tract infections, secondary meningitis, infective endocarditis, and wound infections, and is the cause of outbreaks mainly due to its antimicrobial resistance patterns. The use of broad-spectrum antibiotic coverage with carbapenems is essential in the hospital setting. Therefore, carbapenem-resistant *Acinetobacter baumannii* (CRAB) poses as a very challenging pathogen. *Acinetobacter radioresistens, *a rare species in comparison to the more prevalent *Acinetobacter baumannii*, is an underestimated agent in causing nosocomial infections and also is a potential disseminator of resistance genes. It is also resistant to gamma radiation at 4-8 times higher than other *Acinetobacter *spp*.* and is the source of the class D OXA-23 carbapenemase that can confer carbapenem resistance. Therefore, immediate and precise identification of *A. radioresistens *is crucial for the clinical management of multidrug-resistant bacteremia.

## Introduction

Although this strain was taxonomically described in 1986 after being first isolated from soil and cotton, research has primarily focused on *Acinetobacter baumannii* due to its increasing prevalence as a hospital-acquired infection that is multi-resistant to drug treatment [1,2,3].

As of 2020, a literature search revealed only seven case reports describing eight patients across the world infected with *Acinetobacter radioresistens*. This lesser-discussed member of the *Acinetobacter* family found on the human skin is considered to be a regular part of the human flora; however, rarely is it discovered to be the cause of bacteremia or pneumonia in patients [4]. Here, we report a case of community-acquired *A. radioresistens* bacteremia in an immunocompetent host, identified through the use of matrix-assisted laser desorption/ionization-time of flight (MALDI-TOF).​

## Case presentation

An 83-year-old Caucasian male was brought into an emergency department of a community hospital in Chicago, IL, with suspected community-acquired pneumonia (CAP), following a rapid onset fever and altered mental status. Later in the day, the patient had one episode of emesis and aspirated on his phlegm. The patient had no recent hospitalizations and had been vaccinated for COVID-19. The patient’s past medical history was significant for congestive heart failure (CHF) with preserved left ventricular ejection fraction, atrial fibrillation on Eliquis, and coronary artery disease (CAD) status post coronary artery bypass graft (CABG) in 2016. On presentation, the patient was febrile at 39.0°C, hypotensive at 91/49 mmHg, and tachycardic in the 110s, with an oxygen saturation (SpO_2_) of 94% on 2 L/minute nasal cannula oxygen. On physical examination, the patient had bilaterally decreased breath sounds and an irregular rhythm at 120 beats per minute (bpm).

**Figure 1:**
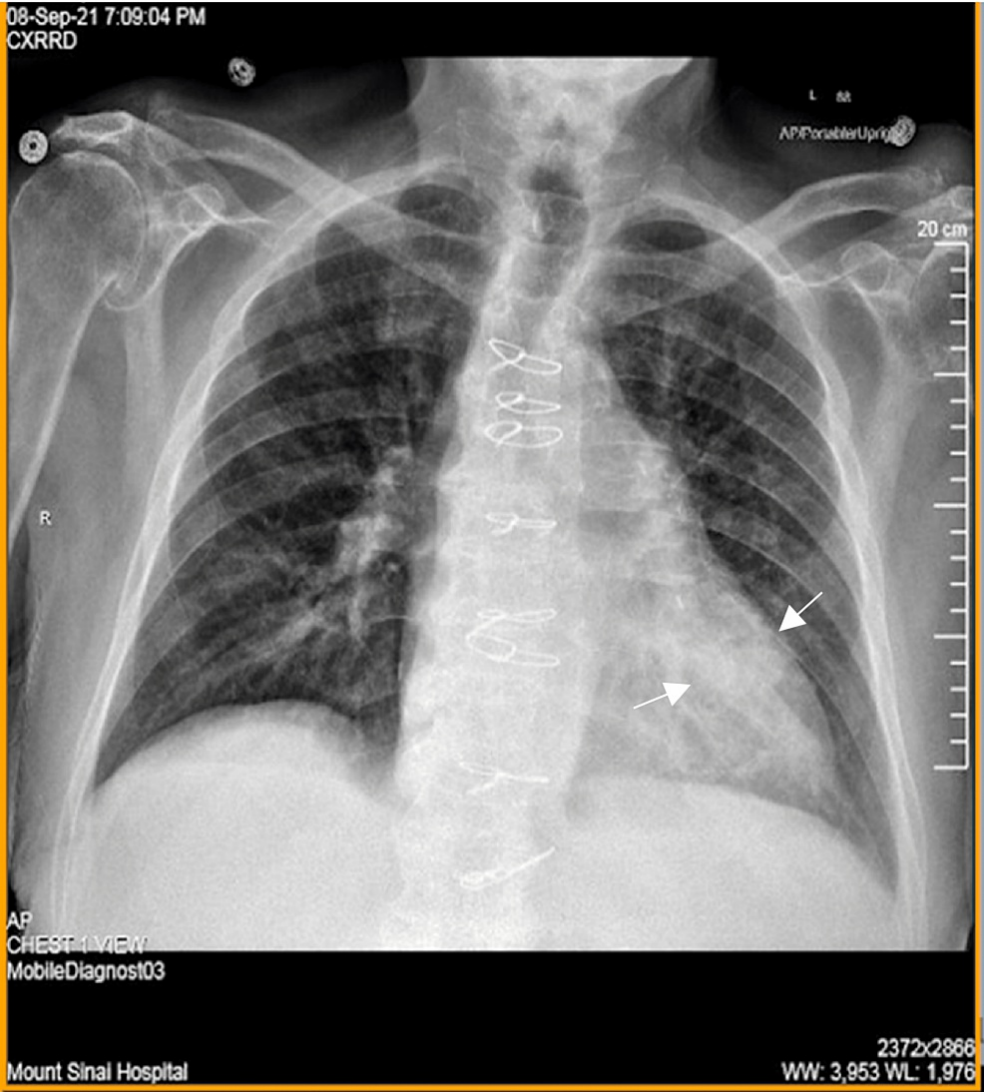
Chest radiograph on admission The opacity at the left retrocardiac region (white arrows) is suspicious for left lower lobe pneumonia versus atelectasis

**Figure 2:**
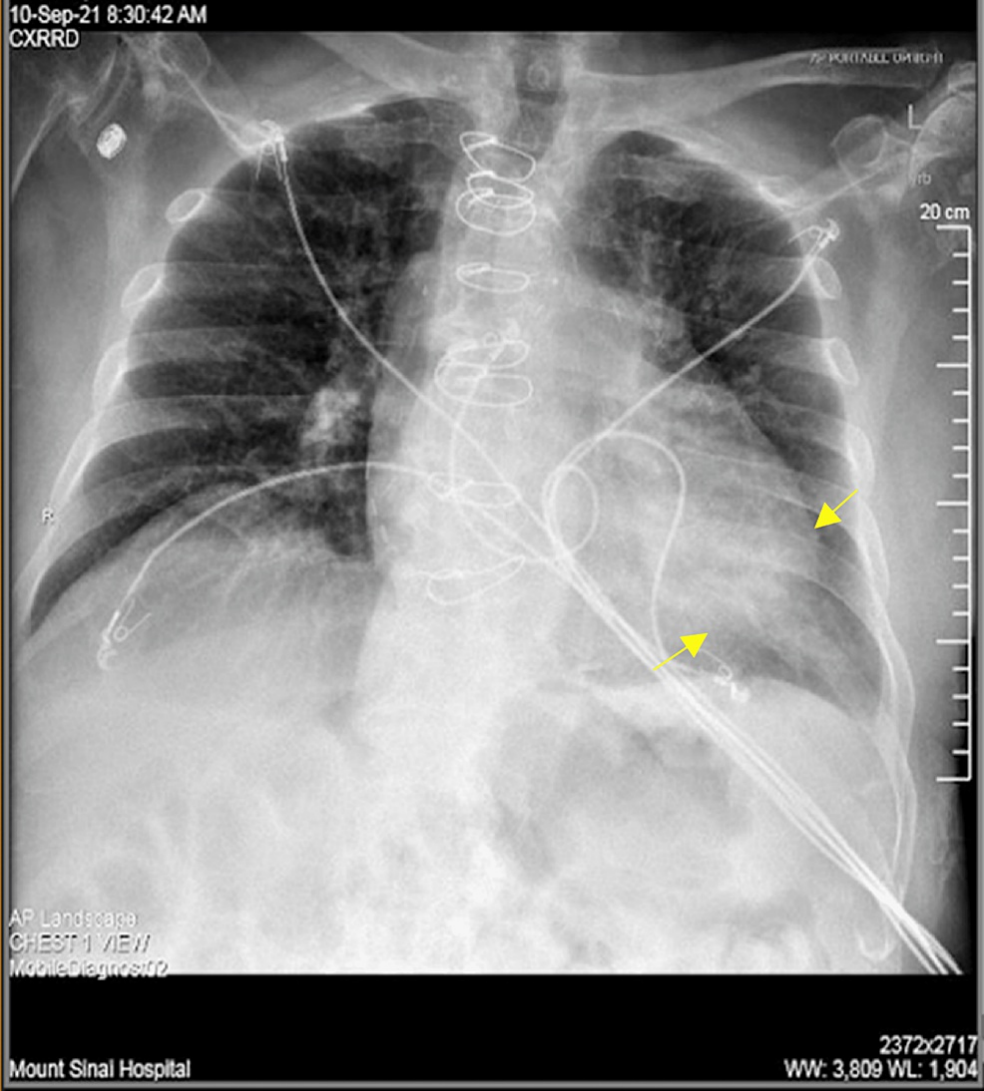
Chest radiograph When compared with the previous study, there is mildly improved aeration at the left lung base (yellow arrows)

Chest X-ray (Figure 1) demonstrated lower lobe consolidation concerning left lower lobe pneumonia. Laboratory findings revealed leukocytosis of 14.1 K/mm^3 ^and lactic acidosis of 2.3 mmol/L. Two blood sample sets from two different sites were sent upon admission, and subsequently, immediate fluid resuscitation and antibiotic therapy were initiated with good response. Furthermore, polymerase chain reaction (PCR) testing for nucleic acid amplification test (NAAT) of SARS-CoV-2 (COVID-19) yielded negative results. All four vials showed the presence of a gram-negative bacillus-shaped organism later identified as *Acinetobacter radioresistens*. Further analysis revealed pan-susceptibility to carbapenems, cephalosporins, aminoglycosides, fluoroquinolones, and trimethoprim/sulfamethoxazole. Urinalysis and urine cultures yielded no significant results, and the patient was started on empiric treatment with IV ceftriaxone 2 g and IV azithromycin 500 mg daily and was admitted for further management. Surveillance blood cultures were negative. Following the resolution of infiltrates seen on day two of admission (Figure 2) with clinical improvement, initial antibiotic therapy was de-escalated to oral amoxicillin/clavulanate 875 mg one tablet every 12 hours for 10 days, which was completed upon discharge. The patient was lost to follow-up. 

## Discussion

Recent data supports the growing evidence of *Acinetobacter* species playing an increasing role in hospital-acquired infections, although limited data exists showing the prevalence of *Acinetobacter radioresistens* within the urban population, as well as its potential to cause sepsis in immunocompetent individuals. To our knowledge, this is the ninth case of *Acinetobacter radioresistens* infection and the fourth case of a successfully treated infection (Table 1).

**Table 1:** Acinetobacter radioresistens clinical case descriptions ^a^Dosing information not provided DM: diabetes mellitus; ESRD: end-stage renal disease; COPD: chronic obstructive pulmonary disease; CHF: congestive heart failure; CAD: coronary artery disease; HCV: hepatitis C virus; HIV: human immunodeficiency virus; NYHA: New York Heart Association; CD4: cluster of differentiation 4; NA: not available

Case	Age	Sex	Comorbid conditions	Source	Treatment	Outcome
Wang et al. [5]	71	F	Adenocarcinoma of the lung	Blood; tracheobronchial	Ampicillin-sulbactam 3 g every six hours	Died
Lopes et al. [6]	73	M	Alzheimer’s disease; Parkinson’s disease	Blood; bronchial	Unknown	Unknown
Visca et al. [7]	32	F	HIV (CD4 cell count 309/mm^3^; viral load <80 copies/mL)	Blood	Ciprofloxacin 400 mg twice a day for 14 days	Survived
Tan et al. [8]	55	M	N/A	Blood; bronchial	Ampicillin-sulbactam 3 g every six hours	Unknown
Brady et al. [9]	53	F	Li-Fraumeni syndrome	Blood	Ampicillin-sulbactam^a^	Survived
	60	M	DMandESRDon hemodialysis	Blood	Cefepime^a^	Survived
Savov et al. [10]	85	M	COPD and CHF (NYHA functional classes III-IV)	Tracheobronchial	Ceftriaxone 2 × 1 g and gentamicin 80 mg	Died
Verma et al. [11]	61	M	COPD and HCV (1.8 million IU/mL viral load <15)	Blood	Ampicillin-sulbactam^a^	Unknown
Our case	83	M	CHF, CAD, and COPD	Blood	Ceftriaxone 2 g and azithromycin 500 mg	Survived

The first case reported an immunocompromised patient with human immunodeficiency virus (HIV) with the combination of neutropenia who survived, while seven isolated cases of immunocompetent individuals, of which four had community-acquired pneumonia, showed resolution on antibiotic therapy [6,7]. A similar case was reported in 2019 in which a middle-aged immunocompetent male was hospitalized due to a severe pulmonary infection and yielded blood cultures isolating *Acinetobacter radioresistens* via matrix-assisted laser desorption/ionization-time of flight mass spectrometry (MALDI-TOF MS) with pan-susceptibility [8].

In our case, *A. radioresistens* was a cause of bacteremia and CAP identified through the use of MALDI-TOF mass spectrometry (MALDI Biotyper CA system, Alverno Laboratories, Hammond, IN), which demonstrated pan-susceptibility to all antibiotics tested (amikacin, ampicillin/sulbactam, cefepime, gentamicin, levofloxacin, meropenem, tobramycin, and trimethoprim/sulfisoxazole). Using MALDI-TOF, this strain of the most fastidious growing bacteria has been able to be quickly and accurately identified through mass spectrometry [12]. Unique bacterial signals measured during the process allow for a highly accurate identification of species and have started to replace old methods of identification [13]. In addition, bacterial antibiotic susceptibility for our organism was determined with the use of Beckman Coulter MicroScan (Beckman Coulter Inc., Brea, CA) analysis, an automated process allowing rapid antibiotic susceptibility testing with gold-standard accuracy [14].

The isolate from our patient was susceptible to all β-lactams, including carbapenems, which suggest a weak expression of the* blaOXA-23* gene and were possibly correlated with favorable course of the disease. Although *Acinetobacter* *radioresistens *has been isolated on the human skin, the bacteria had successfully managed to colonize within the patients’ blood. Its lack of sterilization by gamma radiation and UV irradiation makes it highly survivable within the hospital environment and can potentiate infection. The cause of bacteremia still remains uncertain among the reported cases of CAP in 2019 throughout the world; however, we can place emphasis on the importance of familiarizing providers to *A. radioresistens* and its expression of resistance genes.

## Conclusions

*Acinetobacter radioresistens* is the progenitor of the *blaOXA-23* gene, a class D carbapenemase that causes resistance in *A. baumannii*. Our isolate was susceptible to all β-lactams, including carbapenems, which suggest a weak expression of the *blaOXA-23* gene; however, it must not be ruled out and requires precise identification of this organism such as MALDI-TOF MS. According to the World Health Organization, the *Acinetobacter* species poses to be one of the greatest threats due to the emerging threat of resistance due to the *blaOXA-23* oxacillinase gene constitutionally expressed at low levels; therefore, prompt identification and treatment are necessary. In conclusion, with data available on only a handful of studies, our study suggests that *Acinetobacter radioresistens* remains a highly competent organism that does not seem to be restricted to the hospital setting and that at any time can develop the challenging issue of pan-drug resistance. Therefore, the increasing emergence of this bacterium in the community setting is possible and warrants further studies focusing on the prevalence of this pathogen. A broad approach to antimicrobial drug therapy and the surveillance of resistance genes is imperative to treatment.
